# Resveratrol Suppresses Annulus Fibrosus Cell Apoptosis through Regulating Oxidative Stress Reaction in an Inflammatory Environment

**DOI:** 10.1155/2021/9100444

**Published:** 2021-09-27

**Authors:** Qunqun Shan, Ning Li, Fan Zhang, Peng Yu, Qingxi Meng

**Affiliations:** ^1^Department of Orthopedics, The 960 Hospital of PLA, Jinan, Shandong, China; ^2^Weifang People's Hospital, Weifang, Shandong, China; ^3^Department of Orthopedics, Eightieth Group Army Hospital of PLA Army, Weifang, Shandong, China; ^4^The 11th Cadre Rest Center of Shandong Military Region, Jinan, Shandong, China

## Abstract

During disc degeneration, the increase of inflammatory cytokines and decrease of disc cell density are two prominent features. Enhanced inflammatory reaction contributes to disc annulus fibrosus (AF) cell apoptosis. In this study, we investigated whether resveratrol can suppress AF cell apoptosis in an inflammatory environment. Rat disc AF cells were cultured in medium with or without tumor necrosis factor-*α* (TNF-*α*). Resveratrol was added along with the culture medium supplemented with TNF-*α*. Caspase-3 activity, cell apoptosis ratio, expression of apoptosis-associated molecules (Bcl-2, Bax, caspase-3, cleaved PARP, and cleaved caspase-3), reactive oxygen species (ROS) content, and the total superoxide dismutase (SOD) activity were measured. Our results showed that TNF-*α* significantly increased caspase-3 activity and AF cell apoptosis ratio and upregulated gene/protein expression of Bax, caspase-3, cleaved caspase-3, and cleaved PARP, whereas it downregulated the expression of Bcl-2. Moreover, TNF-*α* significantly increased ROS content but decreased the total SOD activity. Further analysis demonstrated that resveratrol partly attenuated the effects of TNF-*α* on AF cell apoptosis-associated parameters, decreased ROS content, and increased the total SOD activity in the AF cells treated with TNF-*α*. In conclusion, resveratrol attenuates inflammatory cytokine TNF-*α*-induced AF cell apoptosis through regulating oxidative stress reaction in vitro. This study sheds a new light on the protective role of resveratrol in alleviating disc degeneration.

## 1. Introduction

Low back pain is a debilitating disorder that occurs in ~70% of the population [1]. Intervertebral disc degeneration (IDD) is a main contributor to low back pain and the procession of disc herniation. This kind of disease often affects life quality of adults and has a tremendous socioeconomic impact [2, 3]. The mechanism behind disc degeneration has not been fully elucidated.

The promoted cellular apoptosis is one of the main features of the vast majority of disc degeneration [4]. Several previous studies have showed that excessive apoptosis-induced loss of disc cells plays a key role in the disc degeneration process [5, 6]. Bax, Bcl-2, capsase-3, and caspase-9 are some important parameters to evaluate cell apoptosis [7–9]. Previously, lots of studies have reported an increase in the expression of apoptotic genes (Bax and caspase-3/9) and a decrease in the expression of antiapoptotic gene (Bcl-2) in patients with disc degeneration [10, 11].

The inflammatory response is closely associated with disc degeneration. Increased inflammatory cytokines (i.e., TNF-*α* and IL-1*β*) have been detected at the site of herniated disc tissue [12, 13]. These inflammatory cytokines are produced by either leukocytes or disc cells themselves [14]. Moreover, the expression of TNF-*α* and IL-1*β* increases with aging and degree of disc degeneration in degenerative human discs and animal discs [15, 16]. Previously, several studies have showed that inflammatory cytokine IL-1*β* facilitates disc AF cell apoptosis whereas inhibition of IL-1*β* has been shown to suppress disc cell apoptosis and inhibit disc degeneration [17–20]. Hence, a strategy to inhibit or attenuate inflammation response-induced disc cell apoptosis may be helpful to alleviate disc degeneration.

Resveratrol is a kind of nonflavonoid polyphenol which may treat various disorders, such as cancer, ischemic disease, neurodegenerative disease, and cardiovascular disease [21]. It has also been reported that resveratrol plays some positive effects on disc cell's biology [22–29]. Moreover, resveratrol is reported to protect against IL-1*β*-induced nucleus pulposus cell apoptosis [30]. However, whether resveratrol can inhibit or alleviate disc AF cell apoptosis in an inflammatory environment remains unclear. In the present study, we mainly aimed to investigate the effects of resveratrol on AF cell apoptosis in an inflammatory environment and to investigate the changes of oxidative stress reaction in this process.

## 2. Materials and Methods

### 2.1. AF Cell Isolation and Culture Conditions

Thirty-nine rats (8-10 weeks old) were purchased from the Laboratory Animal Center of the 960 Hospital of PLA, and all animal experiments were approved by the Ethics Committee of the 960 Hospital of PLA. Briefly, after the lumbar discs (L1-L5) were separated under sterile conditions, the peripheral AF tissues were obtained. To isolate the individual AF cells, AF tissues were cut into small pieces and digested by 0.2% type I collagenase for 3-4 hours at 37°C. After the subsequent centrifugation (1000 g/min, 5 minutes, 4°C), the AF cell pellets were cultured in DMEM/F12 medium supplemented with 10% fetal bovine serum (FBS, Gibco, USA) with a refresh of culture medium every three days. AF cells with 2 generation of subculture were used in each experiment. The control AF cells were cultured in medium without TNF-*α* whereas the experimental AF cells were cultured in medium with TNF-*α* (50 ng/mL) for 48 hours. In addition, the AF cells treated with TNF-*α* were incubated with exogenous resveratrol (100 *μ*M) (this concentration was referred to a previous study [28]) to investigate its effects on AF cell apoptosis.

### 2.2. Flow Cytometry Analysis

After the cells (2 × 10^5^ cells/well, 6-well) in each group were attached to the plate and those floated in the supernatant were collected together after 48 hours, they were resuspended in cold binding buffer. The apoptosis ratio was measured using an Annexin V/FITC apoptosis detection kit (Beyotime, China) according to the manufacturer's instructions. According to a previous method, cells that were positively stained with Annexin V/FITC but negatively stained with propidium iodide (PI) were apoptotic cells, and the cells that were both positively stained were necrotic cells [31].

### 2.3. Caspase Activity Analysis

AF cells (2 × 10^5^ cells/well, 6-well) were cultured in respective medium for 48 hours. Then, the cells were washed with sterile phosphate buffer solution (PBS) and lysed with lysis solution for 20 minutes. Subsequently, the prepared protein supernatant was used to measure caspase-3 activity and caspase-9 activity according to the manufacturer's instructions (Beyotime, China).

### 2.4. Reactive Oxygen Species (ROS) Content Measurement

AF cells (2 × 10^5^ cells/well, 6-well) were cultured in respective medium for 48 hours. Then, AF cells were treated by 10 *μ*M DCFH-DA for 20 minutes at 37°C, followed by 2 times of washing with serum-free medium. Finally, the fluorescence intensity (excitation/emission: 490/585 nm) indicating ROS content was measured using a fluorescence microplate.

### 2.5. Total Superoxide Dismutase (SOD) Activity Measurement

After AF cells (2 × 10^5^ cells/well, 6-well) were cultured in respective medium for 48 hours, they were lysed using the SOD sample preparing solution, and the protein concentration of the supernatant was measured using a BCA Protein Assay Kit (Beyotime, China). Then, total SOD activity was quantitatively analyzed (U/mg protein) using a Total Superoxide Dismutase Assay Kit with WST-8 Method (Beyotime, China).

### 2.6. Real-Time Polymerase Chain Reaction (PCR) Analysis

After total RNA were extracted using an RNA Sample Total RNA Kit (Tiangen, Beijing, China) and reverse-transcribed into cDNA using a RevertAid First Strand cDNA Synthesis Kit (Thermo Fisher Scientific, USA), real-time PCR was performed via a system containing cDNA, primers, and SYBR Green Mix (DONGSHENG, China). Glyceraldehyde-3-phosphate dehydrogenase (GAPDH) was used as an internal control in this study. The primer sequences were as follows: caspase-3: GTACAGAGCTGGACTGCGGTATTG (forward), AGTCGGCCTCCACTGGTATCTTC (reverse); Bcl-2: ACGGTGGTGGAGGAACTCTTCAG (forward), GGTGTGCAGATGCCGGTTCAG (reverse); and Bax: CCAGGACGCATCCACCAAGAAG (forward), GCTGCCACACGGAAGAAGACC (reverse). Finally, relative expression of Bcl-2, Bax, and caspase-3 was analyzed by the method of 2^−*ΔΔ*CT^.

### 2.7. Western Blot Analysis

After total proteins were extracted and quantified using a BCA Protein Assay Kit, equal protein samples in each group were sequentially separated by sodium dodecyl sulfate-polyacrylamide gel electrophoresis (SDS-PAGE), transferred to the polyvinylidene fluoride (PVDF) membrane, and incubated with primary antibodies (cleaved caspase-3: Cell Signaling Technology, #9664; cleaved PARP: Cell Signaling Technology, #9545) and secondary antibodies according to the standard process. The immunoreactive bands on the PVDF membranes were detected using an enhanced chemiluminescence system (EMD Millipore, Billerica, MA, USA), and the band intensity was quantified using the ImageJ software.

### 2.8. Statistical Analysis

All values were expressed as the mean ± standard deviation. Statistical analyses were performed using SPSS 17.0 software (SPSS, Inc., Chicago, IL, USA). The one-way analysis of variance was used to analyze the significance of differences between these groups. *p* < 0.05 indicated a statistical difference.

## 3. Results

### 3.1. Cell Apoptosis Ratio

**Figure 1:**
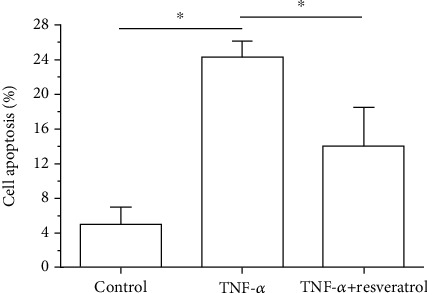
Analysis of annulus fibrosus (AF) cell apoptosis. Data are expressed as mean ± SD, *n* = 3. ^∗^A significant difference (*p* < 0.05) between two groups.

In the TNF-*α* group, AF cell apoptosis ratio was significantly increased compared with that in the control group. When resveratrol was added into the medium of the TNF-*α* group, AF cell apoptosis ratio was partly decreased (Figure 1).

### 3.2. Caspase-3 and Caspase-9 Activity

**Figure 2:**
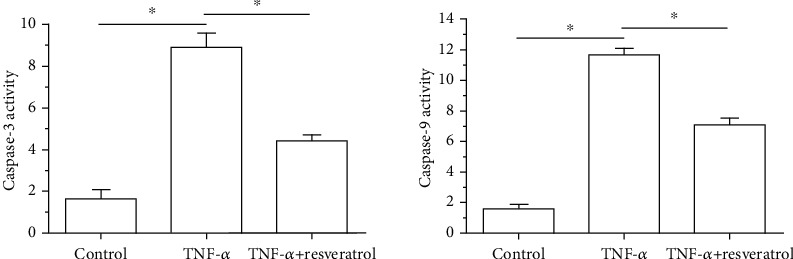
Measurement of caspase-3 and caspase-9 activity of annulus fibrosus (AF) cells. Data are expressed as mean ± SD, *n* = 3. ^∗^A significant difference (*p* < 0.05) between two groups.

In the TNF-*α* group, both caspase-3 activity and caspase-9 activity were significantly increased compared with those in the control group. However, addition of resveratrol in the TNF-*α* group partly decreased both caspase-3 activity and caspase-9 activity (Figure 2).

### 3.3. ROS Content

**Figure 3:**
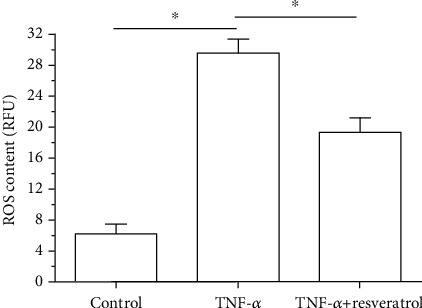
Measurement of reactive oxygen species (ROS) of annulus fibrosus (AF) cells. Data are expressed as mean ± SD, *n* = 3. ^∗^A significant difference (*p* < 0.05) between two groups.

In the TNF-*α* group, ROS content was significantly increased compared with that in the control group, whereas resveratrol could partly decrease ROS content in the TNF-*α* group (Figure 3).

### 3.4. Total SOD Activity

**Figure 4:**
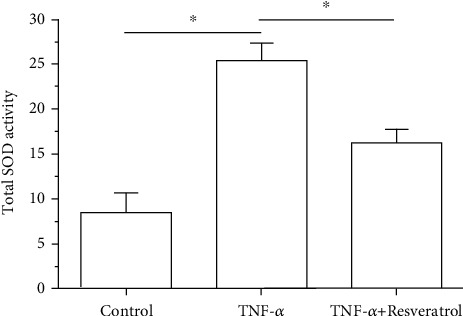
Measurement of total superoxide dismutase (SOD) activity of annulus fibrosus (AF). Data are expressed as mean ± SD, *n* = 3. ^∗^A significant difference (*p* < 0.05) between two groups.

Total SOD activity in the TNF-*α* group was significantly decreased compared with that in the control group; however, addition of resveratrol in the TNF-*α* group could partly increase the total SOD activity (Figure 4).

### 3.5. Gene Expression of Apoptosis-Related Molecules

**Figure 5:**
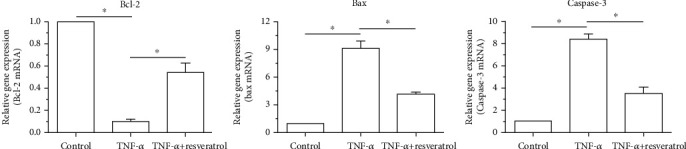
Gene expression of apoptosis-related molecules (Bcl-2, Bax, and caspase-3). Data are expressed as mean ± SD, *n* = 3. ^∗^A significant difference (*p* < 0.05) between two groups.

Results showed that mRNA expression of antiapoptotic genes (Bcl-2) in the TNF-*α* group was significantly downregulated but that of proapoptotic genes (Bax and caspase-3) was significantly upregulated compared with that in the control group. However, addition of resveratrol in the TNF-*α* group partly upregulated mRNA expression of antiapoptotic gene (Bcl-2) and downregulated mRNA expression of proapoptotic genes (Bax and caspase-3) (Figure 5).

### 3.6. Protein Expression of Apoptosis Markers

**Figure 6:**
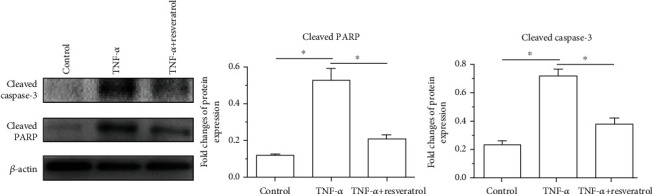
Protein expression of apoptosis-related molecules (cleaved caspase-3 and cleaved PARP). Data are expressed as mean ± SD, *n* = 3. ^∗^A significant difference (*p* < 0.05) between two groups.

Results showed that protein expression of apoptotic markers (cleaved caspase-3 and cleaved PARP) in the TNF-*α* group was significantly increased compared with that in the control group. However, addition of resveratrol in the TNF-*α* group partly decreased protein expression of these apoptotic markers (cleaved caspase-3 and cleaved PARP) (Figure 6).

## 4. Discussion

Intervertebral disc degeneration is a leading cause of low back pain. During disc degeneration, disc cell viability and biosynthesis behaviors often exhibit drastic alterations due to the adverse external or internal niches, which ultimately affect disc structural integrity [32]. The loss of disc cells due to excessive cell apoptosis has been proved to play an important role in mediating disc degeneration [33]. Therefore, it is required to study the cause of disc cell apoptosis.

Enhanced inflammation response is a classical feature during disc degeneration. Inflammatory processes exacerbated by several cytokines (i.e., TNF-*α* and IL-1*β*) are key mediators of disc degeneration and the resultant low back pain [34]. Moreover, inflammation response is closely related with disc cell apoptosis. Previous studies have showed that inflammatory cytokine induces disc cell apoptosis [19, 35]. In this study, we also found that AF cell apoptosis ratio, caspase-3/9 activity, and expression of proapoptotic molecules (Bax, caspase-3, cleaved caspase-3, and cleaved PARP) in the TNF-*α* group were significantly increased compared with those in the control group, confirming that inflammatory cytokine TNF-*α* can induce disc AF cell apoptosis. This is in line with the previous studies [18, 19, 35]. Hence, inhibiting inflammation response or inhibiting the inflammatory response-induced disc cell apoptosis may be an effective strategy to retard disc degeneration process.

Resveratrol is supposed to have efficacy in treating various disorders, such as cancer, ischemic disease, neurodegenerative disease, and cardiovascular disease [21]. Recently, it has also showed that resveratrol may play protective effects on disc cell biology under certain stimulation. Gao et al. have demonstrated resveratrol can enhance matrix biosynthesis of disc nucleus pulposus cells through activating autophagy under oxidative damage [22]. Zhang et al. have showed that resveratrol attenuates mechanical compression-induced disc nucleus pulposus cell apoptosis through regulating the ERK1/2 signaling pathway [29]. Wang et al. have showed that resveratrol attenuates high glucose-induced disc nucleus pulposus cell apoptosis and senescence through activating the ROS-mediated PI3K/Akt pathway [25]. Wang et al. have reported that resveratrol attenuates TNF-*α*-induced MMP-3 expression in human nucleus pulposus cells by activating autophagy via the AMPK/SIRT1 signaling pathway [26]. Importantly, Jiang et al. have found that resveratrol is able to attenuate IL-1beta-mediated disc nucleus pulposus cell apoptosis [30]. However, there is not much more information about the effects of resveratrol on disc AF cells. In this study, we found that addition of resveratrol in the TNF-*α* group partly decreased AF cell apoptosis ratio and caspase-3/9 activity, downregulated expression of proapoptotic genes (Bax, caspase-3, cleaved caspase-3, and cleaved PARP), and upregulated expression of antiapoptotic gene (Bcl-2), indicating that resveratrol can attenuate inflammatory cytokine TNF-*α*-induced disc AF cell apoptosis. To some extent, our results are consistent with the above described studies.

Oxidative stress is believed to be an important step in mediating disc degeneration [36]. ROS has been well recognized as a product of normal cellular mitochondrial metabolism [37]. However, the excessive ROS production will produce detrimental effects on cell biology. A previous study has reported that ROS is elevated in the degenerative disc tissue [38]. Moreover, ROS is a main cause of the increased incidence of cellular apoptosis [39]. In this study, we measured ROS content and the total SOD activity to evaluate the homeostasis of oxidative stress reaction. We found that ROS content was increased while the total SOD activity was decreased in the TNF-*α* group compared with that in the control group. In addition, addition of resveratrol in the TNF-*α* group partly decreased ROS content and increased the total SOD activity. These results suggest that resveratrol can attenuate TNF-*α*-induced oxidative stress injury in AF cells. Taking the corresponding changes of concomitantly happed AF cell apoptosis, we deduced that resveratrol can attenuate AF cell apoptosis through inhibiting oxidative stress damage in an inflammatory environment.

This study also has several limitations. First, because this study is just a preliminary work, we just finished it in vitro, and an in vivo study was not performed to verify the protective effects of resveratrol. Second, there may a dosage effect of resveratrol on inflammation-induced AF cell apoptosis according to our results, but we did not further evaluate this in the present study. To make our study more scientific, these limitations need to be further resolved in the future research.

## 5. Conclusion

In conclusion, we investigated the effects of resveratrol on AF cell apoptosis in an inflammatory environment and studied the role of oxidative stress reaction in this process. Our results demonstrate that resveratrol is effective in attenuating TNF-*α*-induced disc AF cell apoptosis and that this may be mediated through alleviating the oxidative stress injury. This study sheds a new light that resveratrol can suppress TNF-*α*-induced disc AF cell apoptosis through regulating oxidative stress reaction and provides that resveratrol may be a potential drug to retard progression of disc degeneration.

## Data Availability

All data are included in the article.
